# Bridging the gaps: advanced techniques to unlock lipid function in plant reproductive development

**DOI:** 10.1007/s00497-025-00532-2

**Published:** 2025-11-10

**Authors:** Ze-Hua Guo, Mee-Len Chye

**Affiliations:** 1 School of Biological Sciences, The University of Hong Kong, Pokfulam, Hong Kong, China; 2 School of Agriculture and Biology, Shanghai Jiao Tong University, Shanghai, 200240 China; 3 Centre for Agricultural and Food Research, Faculty of Science, Universiti Tunku Abdul Rahman, Jalan Universiti, Bandar Barat, 31900 Kampar, Perak Malaysia

**Keywords:** Acyl-CoA thioesters, Fatty acid biosynthesis, Fatty acid composition, Isothermal titration calorimetry, Pollen germination, Sporopollenin

## Abstract

In plant cells, lipids serve various roles facilitating membrane bilayer formation, energy storage and signaling molecules. Acyl lipids are the most common in distinct plant cell compartments. Lipids regulated by key genes encoding fatty acid desaturases, diacylglycerol acyltransferase, 3-ketoacyl-CoA synthase and acyl-CoA-binding proteins (ACBPs) are deemed crucial during floral development. ACBPs, along with long-chain acyl-CoA synthase, acetyl-CoA carboxylase, fatty acid synthase, acyl-acyl carrier protein desaturases, acyl-ACP thioesterases and the ATP-binding cassette transporter subfamily A, contribute to fatty acid (FA) production, lipid transport and seed oil accumulation, making them bioengineering targets. To investigate lipid function, it is important to use appropriate analytical strategies because different lipid classes contain distinct FA patterns. These well-developed techniques include advanced lipidomic studies using multi-dimensional liquid chromatography-mass spectrometry, matrix-assisted laser desorption/ionization mass spectrometry imaging, lipid-binding assays and x-ray crystallography. As these techniques continue to evolve, further updates on lipid function are expected to rapidly materialize.

## Key message

Plant reproductive organs rely on crucial lipid functions.

## Introduction

Food security is a critical concern worldwide due to a growing population and an increased demand for sustainable agriculture (FAO 2024). In this context, research on plant reproduction plays a pivotal role in generating fruits and seeds, thereby directly impacting crop yield and quality. Some fruits and seeds produce oil, a vital nutrient as people worldwide consume over three quarters of their daily total lipid intake from plant-based oils to cover the essential fatty acids (FAs) that the human body cannot synthesize (Sumara et al. 2023). Also, plant oils support the production of pharmaceuticals and cosmetics (Munir et al. 2017; Raygan et al. 2019). In terms of biochemistry, these oils are extremely complex matrices because they contain chemical compounds that differ in concentration and origin (Sumara et al. 2023). For example, the discovery of bioactive compounds in oils has led to their applications for health benefits and industrial promotion of pressing oils from non-typical oil plants.

Besides its noteworthy role in agriculture, plant reproduction is important for both the persistence and propagation of plant species (Yadava et al. 2019). This stage in the life cycle of the plant is complex and the developmental processes determining seed yield are highly orchestrated (Pajoro et al. 2014). Tasked with a key role, lipid metabolism significantly affects the development and function of reproductive organs such as pollen, anther, stigma and seed (Fadhli Hamdan et al. 2021; Guo et al. 2022; Ortiz et al. 2020; Vallarino et al. 2023). Over the years, plant biologists have focused on these reproductive structures, employing various research techniques to gain a deeper perception of the underlying mechanisms (Borisjuk et al. 2023).

Through lipid biosynthesis, plants generate a diverse range of lipid species including phospholipids and triacylglycerol (TAG), to facilitate different functions (Ohlrogge and Browse 1995). The amphiphilic nature of phospholipids allows them to form fluid structures in cell membranes, serving as permeable barriers and maintaining membrane integrity (Li-Beisson et al. 2013; Ohlrogge and Browse 1995), as their composition is amenable under stress (Du et al. 2010; Oubohssaine et al. 2024). While various phospholipids maintain membrane fluidity and integrity, lipids within the cell membranes act as ligands to activate signal transduction and mediate signaling pathways (Eyster 2007), where lipid-derived second messengers affect cell metabolism and gene expression (Giusto et al. 2010; Newton et al. 2016). TAGs consist of three esterified FA molecules attached to a glycerol backbone, and represent the most abundant lipids in energy reserve during various biological processes (Li-Beisson et al. 2013), including seedling development (Graham 2008), pollen development (Zhang et al. 2009), pollen–stigma interaction (Wolters-Arts et al. 1998), pollen germination (Hernández et al. 2020; Zienkiewicz et al. 2013) and pollen tube elongation (Hernández et al. 2020). In Arabidopsis, TAG breakdown initiates with TAG lipase-mediated FA and glycerol release (Eastmond 2006), and the resultant free FAs (FFAs) are released into the peroxisome (Hayashi et al. 2002; Zolman et al. 2001) for β-oxidation (Graham 2008). Subsequently, FAs are converted into acetyl-CoA, and utilized for carbohydrate synthesis in seed germination and seedling development via the glyoxylate cycle and gluconeogenesis (Graham 2008).

Considering the importance of lipids, a broad range of methods and technologies have been developed to comprehend their function. Following lipid isolation from plants, various lipids including FAs can be derivatized to higher volatility, facilitating analysis by gas chromatography (GC) or GC-mass spectrometry (MS) (Brands et al. 2021). While direct infusion MS (shotgun lipidomics) and liquid chromatography-mass spectrometry (LC–MS) represent high-throughput lipid analyses (Gutbrod et al. 2021), imaging methods based on matrix-assisted laser desorption/ionization mass spectrometry imaging (MALDI-MS) techniques enable locating different lipids at the tissue level (Sturtevant et al. 2021). Also, investigations on lipid-protein interactions are indispensable in systems biology for assigning lipid function in a metabolic background (Guo et al. 2022).

Although the reproductive role of lipids have been previously reviewed in Wan et al. (2020), there is a need for an updated summary that incorporates recent findings and advancements in techniques. In this review, the lipid metabolic pathways in plant reproductive organs are discussed and recent techniques on lipid studies summarized to provide fresh insights..

### Lipids in reproductive organs

#### Lipids function in pollen germination and pollination

For flowering plants, pollen lipids possess various functions including a crucial role during pollen germination. Various FA species including long-chain and very-long chain FAs, are abundant in pollen, and FA composition is known to vary among plants (Breygina et al. 2023; Zhang et al. 2016). Consisting of a mixture of 25 FAs including some omega acids, pollenkitt forms bridges between pollen grains but not between grains and stigma papillae, and performs a key role in pollen protection favoring pollination (Chichiriccò et al. 2019). Pollen development is accompanied by changes in the quantity of lipid metabolites (Rotsch et al. 2017), which are subject to gene regulation during lipid biosynthesis (Table 1). The coordinated action of olive fatty acid desaturases OeFAD2-3 and OeFAD3B led to increases in linoleic and alpha-linolenic acids as observed in germinating pollen, while the action of olive diacylglycerol acyltransferase1 (OeDGAT1) promoted TAG accumulation (Hernández et al. 2020). In Arabidopsis, 3-ketoacyl-CoA synthase4 (AtKCS4) plays a notable role in very-long-chain FA production as an *atkcs4* mutation disrupted pollen tube elongation (Kim et al. 2021). In maize (*Zea mays*), aliphatic metabolism was greatly altered when the contents of lipid constituents especially C16/C18 FAs and their derivatives were significantly reduced in male-sterile mutant *irregular pollen exine2 (ipe2)*, which displayed shrunken anthers and lacked starch accumulation in mature pollen grains (Huo et al. 2020). Two maize mutants (*ms25-6065* and *ms25-6057*) disrupted in fatty acyl reductases (FARs) exhibited defective anther cuticles, abnormal Ubisch body formation, impaired pollen exine formation and total male sterility (Zhang et al. 2021a).

**Table 1:** Genes related to lipid biosynthesis in flowers. The genes involved, the gene family to which each belongs, the organ of its expression, reported function, source and references are summarized in the following table

Genes	Gene family	Organ	Function	Organism	References
*OeFAD2-3* and *OeFAD3B*	Fatty acid desaturase	Pollen	Linoleic and alpha-linolenic acid biosynthesis	Olive	Hernández et al. (2020)
*AtDGAT1*	Diacylglycerol acyltransferase	Pollen	TAG biosynthesis	Arabidopsis	Hernández et al. (2020)
*AtKCS4*	3-ketoacyl-CoA synthase	Pollen	Very-long-chain FA biosynthesis	Arabidopsis	Kim et al. (2021)
*OsC6*	Lipid transfer protein	Pollen	Exchanging lipids between membranes	Rice	Zhang et al. (2010)
*ZmIPE2*	GDSL lipase	Pollen	Lipid breakdown	maize	Huo et al. (2020)
*ZmMs25*	Fatty acyl reductase	Pollen	FA biosynthesis	maize	Zhang et al. (2021a)
*AtLPCAT*	Lysophosphatidylcholine acyltransferase	Pollen	FA biosynthesis	Arabidopsis	Song et al. (2024)
*AtPAH*	Phosphatidic acid phosphohydrolase	Pollen	Lipid breakdown	Arabidopsis	Song et al. (2024)
*AtDGK2* and *AtDGK4*	Diacylglycerol kinase	Pollen	Phospholipid biosynthesis	Arabidopsis	Angkawijaya et al. (2020)
*AtCER1, AtCER3*, *AtCER6* and *AtCER10*	Enoyl-CoA reductase	Stigma	Long-chain and very-long-chain FA biosynthesis	Arabidopsis	Wolters-Arts et al. (1998) and Zheng et al. (2005)
*AtKCS6*	3-ketoacyl-CoA synthase	Stigma	Very-long-chain FA biosynthesis	Arabidopsis	Qin et al. (2022)
*AtCER2-LIKE*	Enoyl-CoA reductase	Anther	Very-long-chain FA biosynthesis	Arabidopsis	Qin et al. (2022)
*AtACBP3*	Acyl-CoA-binding protein	Flower	acyl-lipid homeostasis	Arabidopsis	Guo et al. (2024)
*AtACBP4, AtACBP5* and *AtACBP6*	Acyl-CoA-binding protein	Flower	Wax and cutin biosynthesis	Arabidopsis	Hsiao et al. (2015) and Ye et al. (2017b)
*OsGPAT3*	Glycerol-3-phosphate acyltransferase	Anther	Wax and cutin biosynthesis	Rice	Men et al. (2017)
*OsACOS12*	Acyl-CoA synthetase	Anther	Wax and cutin biosynthesis	Rice	Yang et al. (2017)
*OsDPW2*	Acyl transferase	Anther	Wax and cutin biosynthesis	Rice	Xu et al. (2017)

ACBP, acyl-CoA-binding protein; ACOS, acyl-CoA synthetase; CER, enoyl-CoA reductase; DGAT, diacylglycerol acyltransferase; DGK, diacylglycerol kinase; DPW, defective pollen wall; FAD, fatty acid desaturase; GPAT, glycerol-3-phosphate acyltransferase; IPE, irregular pollen exine; KCS, 3-ketoacyl-CoA synthase; LPCAT, lysophosphatidylcholine acyltransferase; PAH, phosphatidic acid phosphohydrolase; At; *Arabidopsis thaliana*; Oe, *Olea europaea*; Os, *Oryza sativa*; Zm, *Zea mays*

Furthermore, lipids are essential for pollen tube penetration of the stigma and directing pollen-tube growth by regulating water flow to pollen in species with dry and wet stigmas (Wolters-Arts et al. 1998). Pollen tubes of *Nicotiana tabacum* (tobacco) can rapidly remodel their lipidome under heat stress likely by post-transcriptional and/or post-translational regulation (Krawczyk et al. 2022), while pollen sterol content is also subject to environmental conditions (Zu et al. 2021). The double mutant *lpcat pah*, defective in lysophosphatidylcholine acyltransferase (LPCAT) and phosphatidic acid phosphohydrolase (PAH), demonstrated decreased pollen tube growth in the pistil and reduced male transmission in Arabidopsis (Song et al. 2024). Lastly, two Arabidopsis diacylglycerol kinases, DGK2 and DGK4, were deemed crucial in gametogenesis and the biosynthesis of phosphatidylglycerol and phosphatidylinositol in the endoplasmic reticulum (ER) as the *dgk2-1/– dgk4-1/–* plants were gametophyte lethal (Angkawijaya et al. 2020). Overall, these findings highlight the interactive ability of pollen, the importance of lipids in the progamic phase, and their role in protecting pollen and promoting pollination.

### Plant lipids in pollen development and fertilization

Fertilization, as a fundamental process in sexual reproduction of higher plants, relies on very-long-chain FAs (VLCFAs) and their derivatives for proper pollen hydration and germination (Batsale et al. 2021). While the mutation in the Arabidopsis gene encoding enoyl-CoA reductase, *eceriferum10 (cer10)*, showed lower VLCFA content in shoots, disrupting shoot development and yielding shorter inflorescences and floral organ fusion (Zheng et al. 2005), other *cer* mutants *atcer1, atcer3* and *atcer6* displayed abnormal cuticles and male sterility under low-humidity conditions arising from impaired hydration on stigmas, thus highlighting the importance of lipids in plant reproduction (Wolters-Arts et al. 1998). Plant lipid transfer proteins (LTPs) are small and abundant lipid-binding proteins that have been demonstrated to facilitate lipid exchange across membranes (Zhang et al. 2019). OsC6, an LTP from *Oryza sativa* (rice), was shown to bind 1-pyrenedodecanoic acid, and *osc6* mutant rice plants possessed defective orbicules and pollen exine accompanied by reduction in pollen fertility (Zhang et al. 2010).

Pollen coat lipids form a protective outer barrier and are essential for pollen-stigma interaction (Zhang et al. 2021b). In anthers, the expression of *AtKCS6* and *AtCER2*-like genes in the anther endothelium was reported necessary for the production of ≥ C26 VLCFAs and derivatives which accumulate on mature pollen surfaces, serving as signaling molecules to activate water transfer from papilla cells for pollen hydration (Wattelet-Boyer et al. 2016). On the other hand, FA reduction during maturity is believed to discourage self-incompatible pollen mounting, because FA accumulation in stigmas was critical for compatible pollination (Qin et al. 2022). Interestingly, pollen coat VLCFA lipids, which are required for pollen hydration, were not reported to be strictly species-specific (Zhan et al. 2018).

### Sporopollenin biosynthesis and lipid metabolism in pollen development

Sporopollenin is a highly durable and chemically resistant biopolymer that constitutes the outer walls of pollen grains, playing a crucial role in the protection of genetic material during pollination (Xiao et al. 2025). During sporopollenin biosynthesis, long-chain hydroxylated FAs (HFAs) represent the primary precursors (Ariizumi and Toriyama 2011) which are essential for Arabidopsis tapetal development (Ariizumi and Toriyama 2011; Quilichini et al. 2010). Oil bodies provide a rapid source for the biosynthesis of membrane lipids in mature pollen grains (Piffanelli et al. 1998) and their accumulation in the developing pollen grains initiates after the first pollen mitosis (Evans et al. 1991; Owen and Makaroff 1995; Piffanelli et al. 1997, 1998).

It has been reported that acyl-CoA-binding proteins (ACBPs) are important in lipid biosynthesis and transport during anther cuticle and pollen wall development (Hsiao et al. 2015; Ye et al. 2017b), implying their significance in pollen development (Shi et al. 2015). Null mutations of *atacbp4* and *atacbp5* contain more floral wax and cutin than the wild type (WT), while *atacbp4* and *atacbp4atacbp5* pollen exhibited a decrease in stearic acid but an increase in linoleic acid (Ye et al. 2017b). Pollen in the *atacbp4atacbp5atacbp6* triple mutant was deformed, with fewer and smaller oil bodies (Hsiao et al. 2015; Ye et al. 2017b), suggesting that lipid metabolism was abrogated in the *atacbp4atacbp5atacbp6* triple mutant. These studies showed AtACBP4, AtACBP5 and AtACBP6 play a combinatory role in pollen development, potentially affecting acyl-lipid transport during sporopollenin biosynthesis. Moreover, the recent findings on *AtACBP3* conveyed its role in anther development and revealed the potential correlation between lipid content and anther morphology (Guo et al. 2024).

In rice, glycerol-3-phosphate acyltransferase3 (OsGPAT3) (Men et al. 2017) and acyl-CoA synthetase12 (OsACOS12) (Yang et al. 2017) were found to affect wax and cutin biosynthesis and were both required for anther development and male fertility. Also, rice *DEFECTIVE POLLEN WALL2* (*OsDPW2*) encoding an acyl transferase was deemed necessary for rice pollen development, as its mutant (*osdpw2*) had increased amounts of cutin and waxes (Xu et al. 2017). While numerous functions of lipid-related genes in pollen have been reported (Fig. 2), there are more to be explored for potential roles in tapetum development (Yao et al. 2022) and pollen aperture formation (Zhao et al. 2023), which represent the less understood aspects in pollen development.

### Fatty acid biosynthesis pathways in plant seed oils

**Fig. 1:**
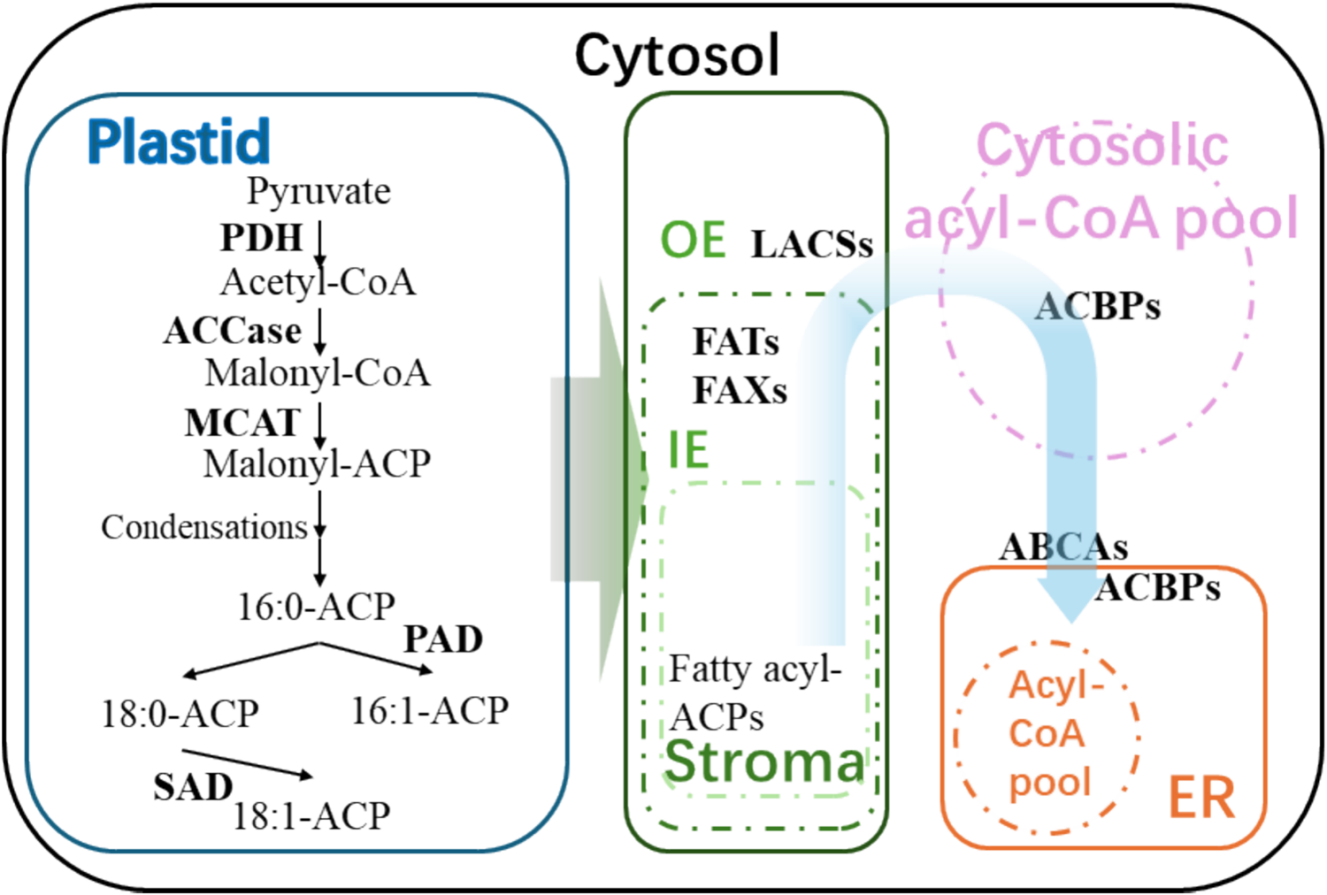
A diagram on de novo fatty acid biosynthesis in seeds. Pathways of lipid biosynthesis are indicated using arrows. The blue box is a representative plastid. The green box indicates a zoom-in view of the plastid stroma, while green boxes within (with dashed borders) represent the inner envelope (IE) and outer envelope (OE) of the stroma. The thick grey arrow indicates transfer of fatty acyl-ACPs to the stroma. The pink circle with dashed outline indicates the cytosolic acyl-CoA pool. The orange box represents the endoplasmic reticulum (ER), and orange circle within (with dashed border) denotes the acyl-CoA pool in the ER. ABCA, ATP-binding cassette transporter subfamily A; ACBP, acyl-CoA-binding protein; ACCase, acetyl-CoA carboxylase; ACP, acyl carrier protein; FAT, fatty acyl-ACP thioesterases; FAX, FATTY ACID EXPORT; LACS, long-chain acyl-CoA synthase; MCAT, malonyl-CoA:ACBP transacylase; PAD, palmitoyl-ACP desaturases; PDH, pyruvate dehydrogenase complex; SAD, stearoyl-ACP desaturase. Proteins including enzymes are bolded

Plant seed oils can confer beneficial health properties (Yang et al. 2018) given their significant phytochemical content including carotenoids, tocopherols, sterols, phenolic compounds, vitamins and minerals (Erkan et al. 2008). In plant seeds, de novo FA biosynthesis occurs in plastids (Fig. 1), which is catalyzed by the type II fatty acid synthase (FAS) with cooperation with other enzymes and enzymatic complexes such as the pyruvate dehydrogenase complex (PDC), acetyl-CoA carboxylase (ACCase) or the FAS complex (Troncoso-Ponce et al. 2016). At the first step of FA synthesis, ACCase transforms acetyl-CoA and bicarbonate into malonyl-CoA (Ohlrogge and Browse 1995). The malonyl group from CoA is then transferred to acyl carrier protein (ACP) by a malonyl-CoA: acyl carrier protein S-malonyltransferase (MCAT) to assemble FAs (Ohlrogge and Browse 1995). Through a series of condensation with acyl-CoA catalyzed by FAS enzymes, the malonyl-ACP is elongated to saturated FA 16:0-ACP (Ohlrogge and Browse 1995). The fate of 16:0-ACP is either desaturation to 16:1-ACP or elongation to 18:0-ACP, which eventually undergoes desaturation to form 18:1-ACP (Ohlrogge and Browse 1995).

## Lipid biosynthesis in seeds

Many genes expressed in seeds have been reported to modulate lipid biosynthesis (Table 2). ​​This process is primarily governed by the coordinated action of multiple enzymes in the plastidial FAS pathway, including ACCase which catalyzes the initial carboxylation step, and the Kennedy pathway which is responsible for triacylglycerol assembly in the endoplasmic reticulum (Ohlrogge and Browse 1995).​​ For instance, the soluble acyl-acyl carrier protein desaturases (AADs) in stroma determines the proportion of newly-synthesized saturated versus monounsaturated fatty acids (Kachroo et al. 2007). There are seven genes encoding AADs in the Arabidopsis genome including FATTY ACID BIOSYNTHESIS2 (AtFAB2) and SUPPRESSOR OF SALICYLIC ACID INSENSITIVE2 (AtSSI2) (Kachroo et al. 2007). Depending on their regio- and substrate-specificity, AADs are characterized as stearoyl-ACP desaturases (SADs) and palmitoyl-ACP desaturases (PADs) (Kachroo et al. 2007). While SADs are responsible for desaturation of 18:0-ACP to 18:1-ACP, PADs catalyze the transition of 16:0-ACP to 16:1-ACP (Kachroo et al. 2007). As the single Arabidopsis *aad* mutation barely affected seed FA composition, it appears that the functions of AADs/SADs are redundant (Kachroo et al. 2007). This is further supported by recent studies on four SAD desaturases (AtFAB2, AtAAD1, AtAAD5 and AtAAD6) which were shown to play collaborative roles in oleic acid biosynthesis in developing Arabidopsis seeds (Kazaz et al. 2020).

**Table 2:** Key gene families associated with lipid biosynthesis in seeds. The genes involved, the gene family to which each belongs, the organ of its expression, reported function, source and references are summarized in the following table

Genes	Gene family	Function	Organism	Reference
*AtFAB2* and* AtSSI2*	Acyl-acyl carrier protein desaturase	Exchanging saturated and unsaturated FAs	Arabidopsis	Kachroo et al. (2007)
*AtAAD1, AtAAD5* and* AtADD6*	Acyl-acyl carrier protein desaturase	Oleic acid biosynthesis	Arabidopsis	Kazaz et al. (2020)
*AtFAT*	Acyl-ACP thioesterase	Biosynthesis of saturated FAs	Arabidopsis	Bonaventure et al. (2003)
*CsFAT*	Acyl-ACP thioesterase	Biosynthesis of saturated FAs	*C. sativa*	Ozseyhan et al. (2018)
*AtLACS1, AtLACS4* and* AtLACS9*	Long-chain acyl-CoA synthase	TAG biosynthesis	Arabidopsis	Zhao et al. (2010) and Jessen et al. (2015)
*BnLACS2*	Long-chain acyl-CoA synthase	TAG biosynthesis	*B. napus*	Ding et al. (2020)
*GmDGAT*	Glycerol-3-phosphate acyltransferase	TAG biosynthesis	*Glycine max*	Zhao et al. (2019)
*AtACBP1*	Acyl-CoA-binding protein	phytosterol biosynthesis	Arabidopsis	Lung et al. (2017 and 2018)
*BnACBP2*	Acyl-CoA-binding protein	TAG biosynthesis	*B. napus*	Liao et al. (2019)
*OsACBP2*	Acyl-CoA-binding protein	Long-chain FA accumulation	Rice	Guo et al. (2019b)
*VfACBP3A* and* VfACBP3B*	Acyl-CoA-binding protein	FA biosynthesis	Tung tree	Pastor et al. (2013)
*EgACBP1* and* EgACBP3*	Acyl-CoA-binding protein	Endosperm oil accumulation	Oil palm	Amiruddin et al. (2020)
*AtABCA9*	ATP-binding cassette transporter subfamily A	Transportation of FAs into the ER	Arabidopsis	Kim et al. (2013)
*AtABCA9*	ATP-binding cassette transporter subfamily A	Seed oil production	*C. sativa*	Cai et al. (2021)

AAD, acyl-acyl carrier protein desaturase; ABCA, ATP-binding cassette transporter subfamily A; ACBP, acyl-CoA-binding protein; FAB2, FATTY ACID BIOSYNTHESIS2 (FAB2); FAT, fatty acyl-ACP thioesterase; FAX, fatty acid export; LACS, long-chain acyl-CoA synthase; SSI2, SUPPRESSOR OF SALICYLIC ACID INSENSITIVE2; At; *Arabidopsis thaliana*; Bn, *Brassica napus;* Cs, *Camelina sativa;* Eg, *Elaeis guineensis;* Os, *Oryza sativa*; Vf, *Vernicia fordii*; Zm, *Zea mays*

### Fatty acid biosynthesis and transportation in seeds

While less than 40% of FAs are trafficked into the plastidic prokaryotic pathway, the majority of de novo synthesized FAs need to be transported to the ER for lipid synthesis via the eukaryotic pathway (Li et al. 2016). Firstly, acyl-ACPs are hydrolyzed by fatty acyl-ACP thioesterases (FAT) at the inner plastid envelope membrane (IE) to FFAs. In Arabidopsis (Bonaventure et al. 2003) and *Camelina sativa* oilseeds (Ozseyhan et al. 2018), it was reported that the loss of function in FAT proteins led to a reduction in saturated FAs. The FATTY ACID EXPORT (FAX) proteins are also FFA transporters anchored in the IE (Li et al. 2015). In Arabidopsis, the *atfax1* mutant possessed lower leaf and floral TAG content (Li et al. 2015), while *atfax2atfax4* seeds contain 30% less TAGs than the WT (Li et al. 2020), providing evidence that loss of AtFAX2 and AtFAX4 function affected FFA transport and TAG biosynthesis during seed development. Following the transportation by FAX, FFAs are mobilized across the plastid outer envelope (OE) via vectorial acylation by long-chain acyl-CoA synthase (LACS), which incorporates FFAs into acyl-CoA thioesters (Fig. 1). In Arabidopsis, nine AtLACS proteins are known to function in lipid biosynthesis. Double mutants of Arabidopsis *atlacs1atlacs9* and *atlacs4atlacs9* showed decreased FA content in seed TAGs (Jessen et al. 2015; Zhao et al. 2010), while the overexpression of *Brassica napus* BnLACS2 in rapeseed resulted in higher seed oil content (Ding et al. 2020).

### Seed acyl-lipid homeostasis and oil accumulation

FFAs are likely transported to the ER by ACBPs (Fig. 1). In Arabidopsis, three cytosolic ACBPs (AtACBP4, AtACBP5 and AtACBP6) have overlapping roles in seed acyl-lipid homeostasis (Hsiao et al. 2014). The single and double mutants of these cytosolic AtACBPs showed altered seed oil composition in comparison to the WT affecting FA and lysophosphatidylcholine (LPC) composition, implying their combinatory function in seed development (Guo et al. 2019a). AtACBP1 negatively affects sterol synthesis, as demonstrated by an increase in total phytosterols in *atacbp1* mutant seeds (Lung et al. 2017, 2018). AtACBP1 has also been suggested to transport membrane-associated acyl-CoAs from the ER to the plasma membrane (Raboanatahiry et al. 2018). In *Brassica*, the high expression of *BnACBP2* during rapid seed oil accumulation implies its potential role in TAG biosynthesis (Liao et al. 2019), and the Class I BnACBP not only modulates C18:1-CoA content but also influences TAG content (Yurchenko et al. 2014). In rice, Class I OsACBP2 is highly expressed in seed embryos, the scutellum and the aleurone layer, with *OsACBP2* overexpression in transgenic rice causing accumulation of long-chain FAs in mature embryos, and enhanced grain size and bran oil content (Guo et al. 2019b). In *Vernicia fordii* (tung tree), both *VfACBP3A* and *VfACBP3B* were reported to be expressed throughout seed development affecting seed FA metabolism (Pastor et al. 2013). In *Elaeis guineensis* (oil palm), EgACBP1 and EgACBP3, also displayed marked increased expression during the active period of endosperm oil accumulation (Amiruddin et al. 2020).

**Fig. 2:**
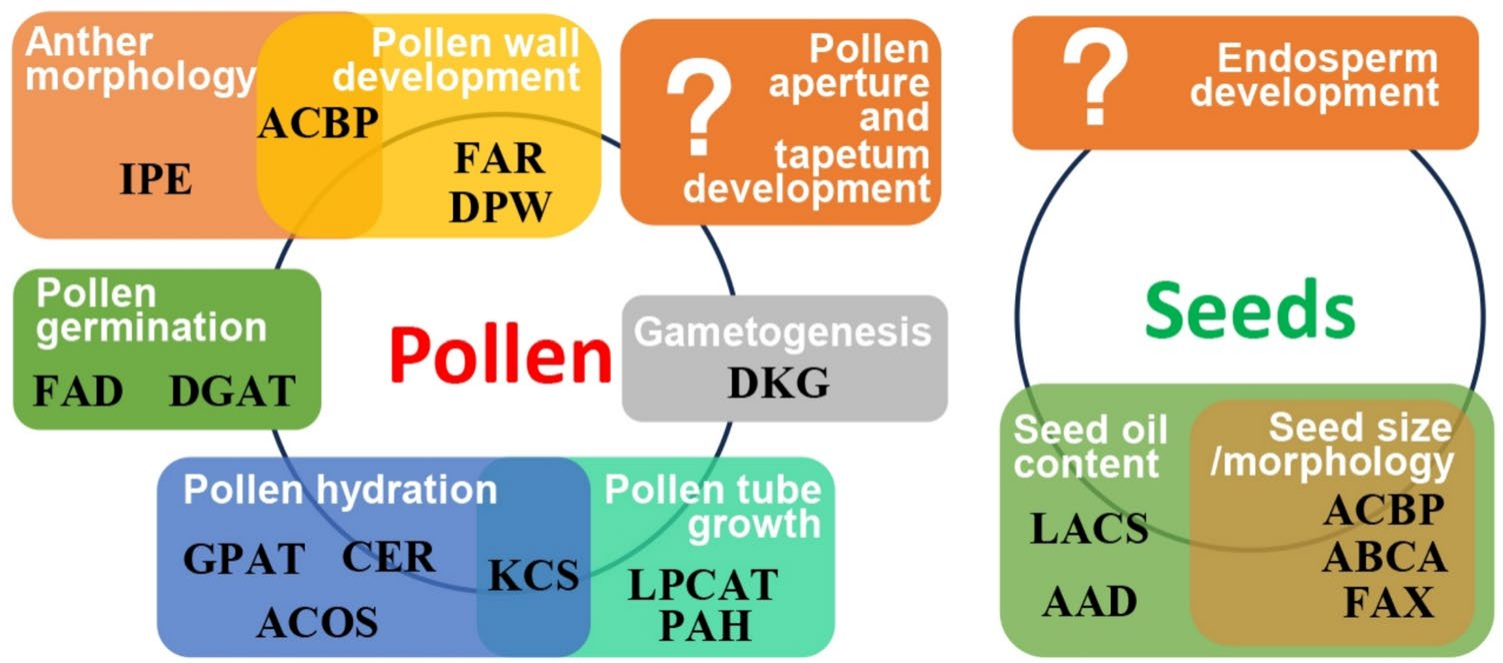
A summary of the proteins in lipid metabolism within reproductive organs. Protein functions in pollen and seeds are depicted. Proteins including enzymes are bolded in black. White letters refer to various events in plant reproduction. The circle on the right indicates biological events in seeds, while that on the left, pollen. The white question marks identify stages for future work on mapping proteins in lipid metabolism to specific functions. AAD, acyl-acyl carrier protein desaturase; ABCA, ATP-binding cassette transporter subfamily A; ACBP, acyl-CoA-binding protein; ACOS, acyl-CoA synthetase; CER, enoyl-CoA reductase; DGAT, diacylglycerol acyltransferase; DGK, diacylglycerol kinases; DPW, defective pollen wall; FAD, fatty acid desaturase; FAR, fatty acyl reductases; FAX, fatty acid export; GPAT, glycerol-3-phosphate acyltransferase; IPE, irregular pollen exine; KCS, 3-ketoacyl-CoA synthase; LACS, long-chain acyl-CoA synthase; LPCAT, lysophosphatidylcholine acyltransferase; PAH, phosphatidic acid phosphohydrolase

Besides ACBPs, the Arabidopsis ATP-binding cassette transporter subfamily A (ABCA) member, AtABCA9, mediates FA transport into the ER (Kim et al. 2013). AtABCA9 was demonstrated to be ER-localized and was highly expressed during seed development (Kim et al. 2013). The *atabca9* mutant seeds were smaller, deformed and contain lower amount of TAGs (Kim et al. 2013). In contrast, TAGs had accumulated in transgenic Arabidopsis AtABCA9-overexpressing seeds (Kim et al. 2013). As the developing *atabca9* seeds incorporated less ^14^C-oleoyl-CoA into TAG than the WT seeds (Kim et al. 2013), AtABCA9 was proposed to transport acyl-CoAs into the ER to provide substrates for TAG synthesis. Also, the overexpression of Arabidopsis AtFAX1 and ABCA9 promoted oil production in *C. sativa* seeds (Cai et al. 2021). Given that the overexpression of lipid-transporting proteins frequently impacted seed oil content, this strategy can be applied to bioengineer and boost seed oil accumulation in transgenic plants. Evolutionary studies have shown that genes involved in seed germination and lipid metabolism undergo strong positive selection, suggesting a potential reservoir of candidate genes for enhancing seed oil content and germination through targeted directional selection (Parakkunnel et al. 2024). The versatility of lipid-related genes suggests that additional functions of these genes have yet to be addressed in other tissues, for instance in seed endosperm (Fig. 2).

Building on the evolutionary insights and tissue-specific potential of lipid-related genes as highlighted in Fig. 2, the next sections explore advanced methodologies to dissect lipid-protein interactions, metabolic pathways, and spatial lipid dynamics. These techniques bridge evolutionary biology with molecular precision: isothermal titration calorimetry (ITC) quantifies lipid-protein binding affinities, X-ray crystallography resolves structural interactions of acyl-CoA-binding proteins (ACBPs) with thioesters, and lipidomic analyses—including optimized extraction protocols and gas chromatography—map lipid composition and flux. Further, enzyme assays and chromatography-mass spectrometry platforms decode acyl-CoA thioester metabolism, while matrix-assisted laser desorption/ionization mass spectrometry imaging (MALDI-MSI) spatially profiles lipids in tissues like the endosperm. Collectively, these approaches illustrate the technology available for enhancing seed germination efficiency and seed oil accumulation, and for translating evolutionary signatures into strategies in bioengineer oil crops in sustainable lipid production.

## Techniques in studying lipids

### Isothermal titration calorimetry offers a robust approach in lipid-protein interaction analysis

To investigate the many functions of newly characterized lipid-related genes in reproduction, several methodologies have gained popularity. In investigations involving the interaction of lipids with proteins, researchers typically employ binding assays. However, traditional methods such as the Lipidex assays (Glatz and Veerkamp 1983) and filter-binding assays (Dowler et al. 2002) have limitations in terms of both efficiency and data accuracy, rendering them more suitable for qualitative analysis of lipid-protein interactions. Isothermal titration calorimetry (ITC) that can measure heat changes generated by binding and record the energetics associated with reactions or processes occurring at a constant temperature (Wiseman et al. 1989), is deemed more appropriate for studying biochemical and molecular interactions (Freire et al. 1990; Jiang et al. 2019)*.* ITC provides information on the thermodynamics of a variety of biophysical process including reactions catalyzed by enzymes and those involving ligand binding that occur between macromolecules, as well as reactions involving ligand- or pH-induced macromolecular conformational changes (Freire et al. 1990). Very early on in the 1970s, ITC was utilized to investigate binding of nucleotides to proteins, antibody to antigen, ligands to human hemoglobin, toxins to proteins as well as peptide-lipid association (Freire et al. 1990), making it fitting for studying protein-lipid binding.

Researchers have employed ITC to analyze the affinity between acyl-CoA thioesters and ACBPs in Arabidopsis (Hsiao et al. 2014; Hu et al. 2018; Xue et al. 2014), rice (Guo and Chye 2021; Guo et al. 2017, 2021) and soybean (Lung et al. 2022). This method has also been applied to address protein–protein interactions between ACBPs and their protein partners (Miao et al. 2019; Ye et al. 2017a). Although traditional binding assays such as the Lipidex assays (Rasmussen et al. 1990) and filter-binding assays (Stevenson et al. 1998) were utilized to study binding involving ACBPs earlier (Chye 1998; Chye et al. 2000; Gao et al. 2010, 2009; Leung et al. 2006; Li et al. 2008; Meng et al. 2011; Xiao and Chye 2011), ITC offers more comprehensive and reliable information by documenting the thermodynamics of binding parameters (Guo et al. 2017, 2021; Hsiao et al. 2014; Hu et al. 2018; Lung et al. 2022; Miao et al. 2019; Xue et al. 2014; Ye et al. 2017a). ITC allows for the accurate determination of thermodynamic parameters, such as changes in Gibbs free energy (ΔG), enthalpy (ΔH) and entropy (ΔS), by using curve-fitting parameters from a selected binding site model (Ladbury et al. 2010). The ΔG value indicates the affinity of binding between the ligand and the protein, while the ΔH value represents the heat energy changes that occur during complex formation at a constant temperature (Ladbury et al. 2010). Furthermore, the equilibrium binding constant (K) can be calculated based on the amount of free or bound ligand at any point during the titration process (Ladbury et al. 2010). These parameters enable the comparison of binding affinities among different proteins and ligands, providing biochemical evidence to support biological functional studies.

### X-ray crystallography: Unveiling ACBP and acyl-CoA thioester interactions

X-ray crystallography has also been used to study protein–ligand interactions, such as those between ACBPs and acyl-CoA thioesters. Using this technique, the acyl-CoA-binding (ACB) domain was shown to be most structurally conserved (Burton et al. 2005), featuring four alpha helices in an up-down-down-up arrangement (Andersen and Poulsen 1993). This unique helical scaffold is preserved across ACBPs from various organisms, including yeast (Teilum et al. 2005), man (Taskinen et al. 2007), armadillo (Costabel et al. 2006), *Plasmodium falciparum* (van Aalten et al. 2001), the plant fungus *Moniliophthora perniciosa* (Monzani et al. 2010) and rice (Guo et al. 2017; Jin et al. 2020).

Moreover, ACBPs have been co-crystallized to demonstrate their interaction with acyl-CoA thioesters (Jin et al. 2020; Kragelund et al. 1993; Monzani et al. 2010; Taskinen et al. 2007). The liganded ACBP modes exhibit diversity: bovine ACBP binding C16:0-CoA esters in a 1 to 1 ratio (Kragelund et al. 1993), human liver ACBPs undergo dimerization when interacting with C14:0-CoA, and *M. perniciosa* ACBPs form dimers when binding C12:0-, C14:0-, C16:0-, C18:0- and C20:0-CoAs (Monzani et al. 2010; Taskinen et al. 2007). Rice OsACBP2 also forms a dimer when binding to its ligand C18:3-CoA, but the spatial conformation differed from human liver ACBPs and the dimerization is suggested to be interchangeable from monomer from size exclusion chromatography-coupled multiangle light scattering analysis (Jin et al. 2020). In summary, x-ray crystallography offers a more detailed perspective on protein-lipid interactions, paving the way for a new field in protein-lipid research.

### Lipidomic analysis: Extraction methods, derivatization for gas chromatography, and advanced mass spectrometry workflow

Lipidomic studies refer to analyses of the lipidome of a tissue, organelle or cell. Their complete profile of lipid species can be accurately derived by modern multi-dimensional LC–MS-based lipidomics (Han 2016). Identifying the lipid alterations can reveal the cellular homeostasis (Harayama and Riezman 2018). The extraction of lipids from tissues is a crucial step for successful lipidomic analysis. The Folch method (Folch et al. 1957) and Bligh and Dyer method (Bligh and Dyer 1959) are classical techniques for lipid extraction, with the former predominantly used for solid tissue and the latter more effective for biological fluids (Pati et al. 2016). Both methods utilize chloroform/methanol in extracting major lipid classes, and lipid extraction from plants mostly rely on the Bligh and Dyer method (Saini et al. 2021). In vegetative tissue, phospholipases can quickly change lipid content, thus it is critical to inactivate this potential lipase activity to ensure successful extraction of plant lipids (Bengtsson et al. 2021).

After derivatization, lipid extracts are analyzed by GC to separate and identify individual compounds in mixtures using a selected column phase based on analyte affinity (Brands et al. 2021; McNair et al. 2019). Recorded by a detector utilizing flame ionization or mass spectrometric methods, the detected data from the column can reveal the composition of the flow-through gas and retention time, defining individual compounds (Bartle and Myers 2002). Absolute quantification of analytes is accomplished by incorporating defined internal standards, aiding in the determination of compound abundance (Brands et al. 2021). Recently, there have been significant advancements in MS techniques, particularly in the areas of data acquisition (Yuan et al. 2024) and MS imaging (Dong and Aharoni 2022; Horn and Chapman 2024; Ma et al. 2023; Yin et al. 2023). These innovations have promoted the application of MS to an even wider range (Yin et al., 2024).

### Acyl-CoA thioester analysis: Enzyme assays, chromatography and mass spectrometry techniques

Acyl-CoA thioesters play essential roles in energy metabolism, the biosynthesis and recycling of complex lipids, posttranslational modification of proteins and the regulation of gene expression (Haslam and Larson 2021). The central role of acyl-CoA thioesters in cellular metabolism is widely known, and many techniques have been developed for the measurement of acyl-CoA content (Haslam and Larson 2021). Analyses are usually carried out by using either enzyme assays of Coenzyme A from hydrolyzed acyl-CoA thioesters, or chromatographic assays of released FAs, or the separation and UV detection of acyl-CoA thioesters by reversed-phase high-performance liquid chromatography (HPLC) (Haslam and Larson 2021).

Specifically for HPLC, the use of solid-phase extraction has improved recovery rates (Golovko and Murphy 2004) and hydrophilic interaction liquid chromatography has been utilized to reduce peak tailing (Abrankó et al. 2018). Moreover, a matrix assisted laser desorption ionization/time-of-flight (MALDI/TOF) mass spectrometer has been reported for complete structural identification of individual acyl-CoAs, including the location of unsaturated bonds in the FA chains (Wang and Hsu 2020). The method developed by Haslam and Larson (2021) represents an invaluable contribution, as it enables the measurement of acyl-CoA content from minute amounts of tissue. This advancement expands the scope of studies on acyl-CoA metabolism to include smaller organs, such as anthers and stigmas, which were previously difficult to analyze due to the limited tissue sample size available. Recent studies in *Leishmania infantum* (Carnielli et al. 2022), *Brassica* (Zhu et al. 2023) and mice (Talamonti et al. 2020) have employed this method to analyze lipid content and composition.

### Matrix-assisted laser desorption/ionization mass spectrometry imaging (MALDI-MSI)

While lipid profiling usually takes homogenized samples, MALDI-MSI can demonstrate spatial localization of lipid molecules, visualizing the unequal or heterogeneously distribution of lipid molecules in different tissues and cell types (Chen et al. 2024b; Sturtevant et al. 2021). In various types of plant tissues, MALDI-MSI has been reported to analyze seed lipid distribution (Lu et al. 2018; Marmon et al. 2017; Sturtevant et al. 2017a), and this technique has been applied for analysis of seeds from Arabidopsis (Sturtevant et al. 2017a), *Gossypium barbadense* (Sturtevant et al. 2017b), *B. napus* (Woodfield et al. 2017), *C. sativa* (Usher et al. 2017), sorghum (Montini et al. 2020) and peanut (Wang et al. 2022). In a standard MALDI-MSI experiment, an intact tissue or tissue section is coated with a chemical matrix, and then subject to the mass spectrometer for a laser beam to pulse over the tissue (Sturtevant et al. 2021). A plume of ions is generated and then examined for their physical properties, resulting in data on physical distribution of ions within a sample (Sturtevant et al. 2021).

The process of preparing plant tissues for MALDI-MSI experiments follows a similar protocol to mammalian tissues (Sturtevant et al. 2021). Despite extra challenges in handling plant tissues, careful handling and preparation can successfully address most technical issues during MALDI-MSI analysis (Sturtevant et al. 2021). Different computing platforms such as MassImage, pyBASIS, and M2aia have enhanced clustering outcomes (Cordes et al. 2021), allowing visualization of sample relationships in MSI research (Zamora Obando et al. 2021). The novel matrix 2,4-dihydroxy-5-nitrobenzoic acid (DHNBA) overcomes previous limitations imposed by MALDI-MSI in plant phytohormone studies such as low hormone abundance and suboptimal matrix performance. DHNBA provides enhanced sensitivity, uniform deposition, and minimal background interference, enabling simultaneous detection, imaging, and precise tracking of multiple hormones (e.g., cytokinins, ABA, IAA, ACC) across diverse tissues to elucidate biosynthesis pathways and distinct functions (Chen et al. 2024a).

## Conclusion and future perspectives

In summary, lipids play a variety of roles in plant cells, including in the formation of the membrane bilayer and acting as energy storage besides signaling molecules (Ohlrogge and Browse 1995). Acyl lipids are the most prevalent group of lipids, representing a wide range of structurally diverse compounds (Li-Beisson et al. 2013; Ohlrogge and Browse 1995). Different lipid classes are present in distinct compartments of plant cells, with each plant tissue displaying a unique lipid profile (Breygina et al. 2023; Erkan et al. 2008).

Lipids play a vital role in floral development, which is regulated by key genes associated with lipid function in flowers include fatty acid desaturases AtFAD2-3, AtFAD3B, AtDGAT1 (Hernández et al. 2020), AtKCS4 (Kim et al. 2021), AtKCS6 and AtCER2-like genes (Qin et al. 2022), as well as ACBPs (Hsiao et al. 2015; Shi et al. 2015; Ye et al. 2017b). Meanwhile, ACBPs, together with LACS (Guo et al. 2022; Jessen et al. 2015; Zhao et al. 2010), ACCase, FAS, AADs (Kachroo et al. 2007; Kazaz et al. 2020; Troncoso-Ponce et al. 2016), FAT, FAX (Bonaventure et al. 2003; Li et al. 2016; Ozseyhan et al. 2018) and ABCA (Kim et al. 2013), play crucial roles in FA production, lipid transport and seed oil accumulation, making them potential targets for bioengineering strategies to increase or modify seed oil content (Cai et al. 2021).

The amount of lipids within the cell can vary depending on their functions, and their quantity can range from highly abundant structural and storage lipids to smaller amounts for signaling lipids (Saini et al. 2021). Numerous molecular species with distinct FA patterns make up various lipid classes, including membrane glycerolipids and TAGs (Ohlrogge and Browse 1995). The abundance of these molecular species within a lipid class is an important factor when determining the analytical strategy for their examination (Saini et al. 2021). These strategies involve advanced lipidomic studies using modern multi-dimensional LC–MS techniques (Han 2016) as well as MALDI-MSI which allows for the spatial localization of lipid molecules (Sturtevant et al. 2021). Additionally, lipid-protein interactions can be investigated through lipid-binding assays and x-ray crystallography techniques (Guo et al. 2022). Given the importance of lipid function, scientists have utilized well-established methods such as strong anion exchange (SAX)-HPLC (Laha et al. 2021) and nuclear magnetic resonance (NMR) spectroscopy (Knaack et al. 2021). With the evolution of these techniques, the functions of lipids are anticipated to be rapidly elucidated.

## Data Availability

No datasets were generated or analysed during the current study.

## References

[CR1] Abrankó L, Williamson G, Gardner S, Kerimi A (2018) Comprehensive quantitative analysis of fatty-acyl-Coenzyme A species in biological samples by ultra-high performance liquid chromatography–tandem mass spectrometry harmonizing hydrophilic interaction and reversed phase chromatography. J Chromatogr A 1534:111–122. 10.1016/j.chroma.2017.12.052 29290399 10.1016/j.chroma.2017.12.052

[CR2] Amiruddin N, Chan PL, Azizi N, Morris PE, Chan KL, Ong PW, Rosli R, Masura SS, Murphy DJ, Sambanthamurthi R (2020) Characterization of oil palm acyl-CoA-binding proteins and correlation of their gene expression with oil synthesis. Plant Cell Physiol 61:735–747. 10.1093/pcp/pcz237 31883014 10.1093/pcp/pcz237

[CR3] Andersen KV, Poulsen FM (1993) The three-dimensional structure of acyl-coenzyme A binding protein from bovine liver: structural refinement using heteronuclear multidimensional NMR spectroscopy. J Biomol NMR 3:271–284. 10.1007/BF00212514 8358232 10.1007/BF00212514

[CR4] Angkawijaya AE, Nguyen VC, Gunawan F, Nakamura Y (2020) A pair of Arabidopsis diacylglycerol kinases essential for gametogenesis and endoplasmic reticulum phospholipid metabolism in leaves and flowers. Plant Cell 32:2602–2620. 10.1105/tpc.20.00251 32471859 10.1105/tpc.20.00251 PMC7401011

[CR5] Ariizumi T, Toriyama K (2011) Genetic regulation of sporopollenin synthesis and pollen exine development. Annu Rev Plant Biol 62:437–460. 10.1146/annurev-arplant-042809-112312 21275644 10.1146/annurev-arplant-042809-112312

[CR6] Bartle KD, Myers P (2002) History of gas chromatography. TrAC. Trends Anal Chem 21:547–557. 10.1016/S0165-9936(02)00806-3

[CR7] Batsale M, Bahammou D, Fouillen L, Mongrand S, Joubès J, Domergue F (2021) Biosynthesis and functions of very-long-chain fatty acids in the responses of plants to abiotic and biotic stresses. Cells 10:1284. 10.3390/cells10061284 34064239 10.3390/cells10061284 PMC8224384

[CR8] Bengtsson JD, Wallis JG, Browse J (2021) Lipid Isolation from Plants. In: Plant Lipids: Methods and Protocols (Springer), pp 3–13 10.1007/978-1-0716-1362-7_1 10.1007/978-1-0716-1362-7_1 PMC8612076 34047968

[CR9] Bligh EG, Dyer WJ (1959) A rapid method of total lipid extraction and purification. Can J Biochem Physiol 37:911–917. 10.1139/o59-099 13671378 10.1139/o59-099

[CR10] Bonaventure G, Salas JJ, Pollard MR, Ohlrogge JB (2003) Disruption of the FATB gene in Arabidopsis demonstrates an essential role of saturated fatty acids in plant growth. Plant Cell 15:1020–1033. 10.1105/tpc.008946 12671095 10.1105/tpc.008946 PMC152346

[CR11] Borisjuk L, Horn P, Chapman K, Jakob PM, Gündel A, Rolletschek H (2023) Seeing plants as never before. New Phytol 238:1775–1794. 10.1111/nph.18871 36895109 10.1111/nph.18871

[CR12] Brands M, Gutbrod P, Dörmann P (2021) Lipid analysis by gas chromatography and gas chromatography–mass spectrometry. In: Plant Lipids: Methods and Protocols (Springer), pp 43–57 10.1007/978-1-0716-1362-7_4 10.1007/978-1-0716-1362-7_4 34047971

[CR13] Breygina M, Voronkov A, Ivanova T, Babushkina K (2023) Fatty acid composition of dry and germinating pollen of gymnosperm and angiosperm plants. Int J Mol Sci 24:9717. 10.3390/ijms24119717 37298668 10.3390/ijms24119717 PMC10253635

[CR14] Burton M, Rose TM, Faergeman NJ, Knudsen J (2005) Evolution of the acyl-CoA binding protein (ACBP). Biochem J 392:299–307. 10.1042/BJ20050664 16018771 10.1042/BJ20050664 PMC1316265

[CR15] Cai G, Wang G, Kim SC, Li J, Zhou Y, Wang X (2021) Increased expression of fatty acid and ABC transporters enhances seed oil production in camelina. Biotech Biofuels 14:1–11. 10.1186/s13068-021-01899-w 10.1186/s13068-021-01899-w PMC7913393 33640013

[CR16] Carnielli JB, Dave A, Romano A, Forrester S, de Faria PR, Monti-Rocha R, Costa CH, Dietze R, Graham IA, Mottram JC (2022) 3′ Nucleotidase/nuclease is required for *Leishmania infantum* clinical isolate susceptibility to miltefosine. EBioMedicine 86:104378. 10.1016/j.ebiom.2022.104378 36462405 10.1016/j.ebiom.2022.104378 PMC9713291

[CR17] Chen L, Zhang Y, Hao Q, Fu J, Bao Z, Bu Y, Sun N, Wu X, Lu L, Kong Z (2024a) Enhancement of in situ detection and imaging of phytohormones in plant tissues by MALDI-MSI using 2, 4-dihydroxy-5-nitrobenzoic acid as a novel matrix. New Phytol 243:2021–2036. 10.1111/nph.19964 39014531 10.1111/nph.19964

[CR18] Chen YJ, Zeng HS, Jin HL, Wang HB (2024b) Applications of mass spectrometry imaging in botanical research. Adv Biotech 2:6. 10.1007/s44307-024-00014-y 10.1007/s44307-024-00014-y PMC11740876 39883274

[CR19] Chichiriccò G, Pacini E, Lanza B (2019) Pollenkitt of some monocotyledons: lipid composition and implications for pollen germination. Plant Biol 21:920–926. 10.1111/plb.12998 31034724 10.1111/plb.12998

[CR20] Chye ML (1998) Arabidopsis cDNA encoding a membrane-associated protein with an acyl-CoA binding domain. Plant Mol Biol 38:827–838. 10.1023/a:1006052108468 9862500 10.1023/a:1006052108468

[CR21] Chye ML, Li HY, Yung MH (2000) Single amino acid substitutions at the acyl-CoA-binding domain interrupt (14) C palmitoyl-CoA binding of ACBP2, an Arabidopsis acyl-CoA-binding protein with ankyrin repeats. Plant Mol Biol 44:711–721. 10.1023/A:1026524108095 11202434 10.1023/a:1026524108095

[CR22] Cordes J, Enzlein T, Marsching C, Hinze M, Engelhardt S, Hopf C, Wolf I (2021) M2aia—Interactive, fast, and memory-efficient analysis of 2D and 3D multi-modal mass spectrometry imaging data. GigaScience 10, giab049 10.1093/gigascience/giab049 10.1093/gigascience/giab049 PMC8290197 34282451

[CR23] Costabel MD, Ermacora MR, Santome JA, Alzari PM, Guerin DMA (2006) Structure of armadillo ACBP: a new member of the acyl-CoA-binding protein family. Acta Crystallogr F 62:958–961. 10.1107/S1744309106038164 10.1107/S1744309106038164 PMC2225200 17012783

[CR24] Ding LN, Gu SL, Zhu FG, Ma ZY, Li J, Li M, Wang Z, Tan XL (2020) Long-chain acyl-CoA synthetase 2 is involved in seed oil production in *Brassica napus*. BMC Plant Biol 20:1–14. 10.1186/s12870-020-2240-x 31931712 10.1186/s12870-020-2240-x PMC6958636

[CR25] Dong Y, Aharoni A (2022) Image to insight: exploring natural products through mass spectrometry imaging. Nat Prod Rep 39:1510–1530. 10.1039/d2np00011c 35735199 10.1039/d2np00011c

[CR26] Dowler S, Kular G, Alessi DR (2002) Protein lipid overlay assay. Sci. STKE 2002 (129): pl6 10.1126/stke.2002.129.pl6 10.1126/stke.2002.129.pl6 11972359

[CR27] Du ZY, Xiao S, Chen QF, Chye ML (2010) Depletion of the membrane-associated Acyl-Coenzyme A-Binding Protein ACBP1 enhances the ability of cold acclimation in Arabidopsis. Plant Physiol 152:1585–1597. 10.1104/pp.109.147066 20107029 10.1104/pp.109.147066 PMC2832255

[CR28] Eastmond PJ (2006) *SUGAR-DEPENDENT1* encodes a patatin domain triacylglycerol lipase that initiates storage oil breakdown in germinating Arabidopsis seeds. Plant Cell 18:665–675. 10.1105/tpc.105.040543 16473965 10.1105/tpc.105.040543 PMC1383641

[CR29] Erkan N, Ayranci G, Ayranci E (2008) Antioxidant activities of rosemary (*Rosmarinus Officinalis* L.) extract, blackseed (*Nigella sativa* L.) essential oil, carnosic acid, rosmarinic acid and sesamol. Food Chem 110:76–82. 10.1016/j.foodchem.2008.01.058 26050168 10.1016/j.foodchem.2008.01.058

[CR30] Evans D, Taylor P, Singh M, Knox R (1991) Quantitative analysis of lipids and protein from the pollen of *Brassica napus* L. Plant Sci 73:117–126. 10.1016/0168-9452(91)90133-S

[CR31] Eyster KM (2007) The membrane and lipids as integral participants in signal transduction: lipid signal transduction for the non-lipid biochemist. Adv Physiol Educ 31:5–16. 10.1152/advan.00088.2006 17327576 10.1152/advan.00088.2006

[CR32] Fadhli Hamdan M, Lung SC, Guo ZH, Chye ML (2021) Roles of acyl-CoA-binding proteins in plant reproduction. J Exp Bot 73:2918–2936. 10.1093/jxb/erab499 10.1093/jxb/erab499 35560189

[CR33] FAO (2024) FAO Cereal Supply and Demand Brief. https://www.fao.org/worldfoodsituation/csdb/en

[CR34] Folch J, Lees M, Stanley GS (1957) A simple method for the isolation and purification of total lipides from animal tissues. J Biol Chem 226:497–509 13428781

[CR35] Freire E, Mayorga OL, Straume M (1990) Isothermal titration calorimetry. Anal Chem 62:A950–A959

[CR36] Gao W, Xiao S, Li HY, Tsao SW, Chye ML (2009) *Arabidopsis thaliana* acyl-CoA-binding protein ACBP2 interacts with heavy-metal-binding farnesylated protein AtFP6. New Phytol 181:89–102. 10.1111/j.1469-8137.2008.02631.x 18823312 10.1111/j.1469-8137.2008.02631.x

[CR37] Gao W, Li HY, Xiao S, Chye ML (2010) Acyl-CoA-binding protein 2 binds lysophospholipase 2 and lysoPC to promote tolerance to cadmium-induced oxidative stress in transgenic Arabidopsis. Plant J 62:989–1003. 10.1111/j.1365-313X.2010.04209.x 20345607 10.1111/j.1365-313X.2010.04209.x

[CR38] Giusto NM, Pasquaré SJ, Salvador GA, de Boschero MGI (2010) Lipid second messengers and related enzymes in vertebrate rod outer segments. J Lipid Res 51:685–700. 10.1194/jlr.R001891 19828910 10.1194/jlr.R001891 PMC2842151

[CR39] Glatz JF, Veerkamp JH (1983) A radiochemical procedure for the assay of fatty acid binding by proteins. Anal Biochem 132:89–95. 10.1016/0003-2697(83)90429-3 6194713 10.1016/0003-2697(83)90429-3

[CR40] Golovko MY, Murphy EJ (2004) An improved method for tissue long-chain acyl-CoA extraction and analysis. J Lipid Res 45:1777–1782. 10.1194/jlr.D400004-JLR200 15210839 10.1194/jlr.D400004-JLR200

[CR41] Graham IA (2008) Seed storage oil mobilization. Annu Rev Plant Biol 59:115–142. 10.1146/annurev.arplant.59.032607.092938 18444898 10.1146/annurev.arplant.59.032607.092938

[CR42] Guo ZH, Chan WH, Kong GK, Hao Q, Chye ML (2017) The first plant acyl-CoA-binding protein structures: the close homologues OsACBP1 and OsACBP2 from rice. Acta Crystallogr D 73:438–448. 10.1107/S2059798317004193 10.1107/S2059798317004193 28471368

[CR43] Guo ZH, Ye ZW, Haslam RP, Michaelson LV, Napier JA, Chye ML (2019a) Arabidopsis cytosolic acyl-CoA-binding proteins function in determining seed oil composition. Plant Direct 3:e00182. 10.1002/pld3.182 31844833 10.1002/pld3.182 PMC6892995

[CR44] Guo ZH, Haslam RP, Michaelson LV, Yeung EC, Lung SC, Napier JA, Chye ML (2019b) The overexpression of rice ACYL-COA-BINDING PROTEIN 2 increases grain size and bran oil content in transgenic rice. Plant J 100:1132–1147. 10.1111/tpj.14503 31437323 10.1111/tpj.14503

[CR45] Guo ZH, Pogancev G, Meng W, Du ZY, Liao P, Zhang R, Chye ML (2021) The overexpression of rice ACYL-COA-BINDING PROTEIN4 improves salinity tolerance in transgenic rice. Enviorn Exp Bot 183:104349. 10.1016/j.envexpbot.2020.104349

[CR46] Guo ZH, Lung SC, Fadhli Hamdan M, Chye ML (2022) Interactions between plant lipid-binding proteins and their ligands. Prog Lipid Res 86:101156. 10.1016/j.plipres.2022 35066006 10.1016/j.plipres.2022.101156

[CR47] Guo ZH, Hu T-H, Hamdan MF, Li M, Wang R, Xu J, Lung SC, Liang W, Shi J, Zhang D, Chye ML (2024) A promoter polymorphism defines distinct roles in anther development for Col-0 and L*er*-0 alleles of Arabidopsis *ACYL-COA BINDING PROTEIN3*. New Phytol 243:1424–1439. 10.1111/nph.19924 38922886 10.1111/nph.19924

[CR48] Guo ZH, Chye ML (2021) Investigations of Lipid Binding to Acyl-CoA-Binding Proteins (ACBP) Using Isothermal Titration Calorimetry (ITC). In: Plant Lipids. Methods in Molecular Biology (Springer), pp 401–415 10.1007/978-1-0716-1362-7_23 10.1007/978-1-0716-1362-7_23 34047990

[CR49] Gutbrod K, Peisker H, Dörmann P (2021) Direct infusion mass spectrometry for complex lipid analysis. In: Plant Lipids: Methods and Protocols (Springer), pp 101–115 10.1007/978-1-0716-1362-7_7 10.1007/978-1-0716-1362-7_7 34047974

[CR50] Han X (2016) Lipidomics for studying metabolism. Nat Rev Endocrinol 12:668–679. 10.1038/nrendo.2016.98 27469345 10.1038/nrendo.2016.98

[CR51] Harayama T, Riezman H (2018) Understanding the diversity of membrane lipid composition. Nat Rev Mol Cell Biol 19:281–296. 10.1038/nrm.2017.138 29410529 10.1038/nrm.2017.138

[CR52] Haslam RP, Larson TR (2021) Techniques for the measurement of molecular species of acyl-CoA in plants and microalgae. In: Plant Lipids: Methods and Protocols (Springer), pp 203–218 10.1007/978-1-0716-1362-7_12 10.1007/978-1-0716-1362-7_12 34047979

[CR53] Hayashi H, De Bellis L, Hayashi Y, Nito K, Kato A, Hayashi M, Hara-Nishimura I, Nishimura M (2002) Molecular characterization of an Arabidopsis acyl-coenzyme a synthetase localized on glyoxysomal membranes. Plant Physiol 130:2019–2026. 10.1104/pp.012955 12481085 10.1104/pp.012955 PMC166713

[CR54] Hernández ML, Lima-Cabello E, Alché JdD, Martínez-Rivas JM, Castro AJ (2020) Lipid composition and associated gene expression patterns during pollen germination and pollen tube growth in olive (*Olea europaea* L*.*). Plant Cell Physiol 61:1348–1364. 10.1093/pcp/pcaa063 32384163 10.1093/pcp/pcaa063 PMC7377348

[CR55] Horn PJ, Chapman KD (2024) Imaging plant metabolism in situ. J Exp Bot 75:1654–1670. 10.1093/jxb/erad423 37889862 10.1093/jxb/erad423 PMC10938046

[CR56] Hsiao AS, Haslam RP, Michaelson LV, Liao P, Chen QF, Sooriyaarachchi S, Mowbray SL, Napier JA, Tanner JA, Chye ML (2014) Arabidopsis cytosolic acyl-CoA-binding proteins ACBP4, ACBP5 and ACBP6 have overlapping but distinct roles in seed development. BioSci Rep 34:e00165. 10.1093/pcp/pcu163 25423293 10.1042/BSR20140139 PMC4274664

[CR57] Hsiao AS, Yeung EC, Ye ZW, Chye ML (2015) The Arabidopsis cytosolic Acyl-CoA-binding proteins play combinatory roles in pollen development. Plant Cell Physiol 56:322–333. 10.1093/pcp/pcu163 25395473 10.1093/pcp/pcu163

[CR58] Hu TH, Lung SC, Ye ZW, Chye ML (2018) Depletion of Arabidopsis ACYL-COA-BINDING PROTEIN3 affects fatty acid composition in the phloem. Front Plant Sci 9:2. 10.3389/fpls.2018.00002 29422909 10.3389/fpls.2018.00002 PMC5789640

[CR59] Huo Y, Pei Y, Tian Y, Zhang Z, Li K, Liu J, Xiao S, Chen H, Liu J (2020) IRREGULAR POLLEN EXINE2 encodes a GDSL lipase essential for male fertility in maize. Plant Physiol 184:1438–1454. 10.1104/pp.20.00105 32913046 10.1104/pp.20.00105 PMC7608179

[CR60] Jessen D, Roth C, Wiermer M, Fulda M (2015) Two activities of long-chain acyl-coenzyme A synthetase are involved in lipid trafficking between the endoplasmic reticulum and the plastid in Arabidopsis. Plant Physiol 167:351–366. 10.1104/pp.114.250365 25540329 10.1104/pp.114.250365 PMC4326746

[CR61] Jiang Z, Zhou X, Tao M, Yuan F, Liu L, Wu F, Wu X, Xiang Y, Niu Y, Liu F, Li C, Ye R, Byeon B, Xue Y, Zhao H, Wang H-N, Crawford BM, Johnson DM, Hu C, Pei C, Zhou W, Swift GB, Zhang H, Vo-Dinh T, Hu Z, Siedow JN, Pei Z-M (2019) Plant cell-surface GIPC sphingolipids sense salt to trigger Ca^2+^ influx. Nature 572:341–346. 10.1038/s41586-019-1449-z 31367039 10.1038/s41586-019-1449-z

[CR62] Jin J, Guo ZH, Hao Q, Chye ML (2020) Crystal structure of the rice acyl-CoA-binding protein OsACBP2 in complex with C18: 3-CoA reveals a novel pattern of binding to acyl-CoA esters. FEBS Lett 594:3568–3575. 10.1002/1873-3468.13923 32888212 10.1002/1873-3468.13923

[CR63] Kachroo A, Shanklin J, Whittle E, Lapchyk L, Hildebrand D, Kachroo P (2007) The *Arabidopsis* stearoyl-acyl carrier protein-desaturase family and the contribution of leaf isoforms to oleic acid synthesis. Plant Mol Biol 63:257–271. 10.1007/s11103-006-9086-y 17072561 10.1007/s11103-006-9086-y

[CR64] Kazaz S, Barthole G, Domergue F, Ettaki H, To A, Vasselon D, De Vos D, Belcram K, Lepiniec L, Baud S (2020) Differential activation of partially redundant Δ9 stearoyl-ACP desaturase genes is critical for omega-9 monounsaturated fatty acid biosynthesis during seed development in Arabidopsis. Plant Cell 32:3613–3637. 10.1105/tpc.20.00554 32958563 10.1105/tpc.20.00554 PMC7610281

[CR65] Kim S, Yamaoka Y, Ono H, Kim H, Shim D, Maeshima M, Martinoia E, Cahoon EB, Nishida I, Lee Y (2013) AtABCA9 transporter supplies fatty acids for lipid synthesis to the endoplasmic reticulum. PNAS 110:773–778. 10.1073/pnas.1214159110 23269834 10.1073/pnas.1214159110 PMC3545803

[CR66] Kim J, Lee SB, Suh MC (2021) Arabidopsis 3-ketoacyl-CoA synthase 4 is essential for root and pollen tube growth. J Plant Biol 64:155–165. 10.1007/s12374-020-09288-w

[CR67] Knaack W, Hölzl G, Gisch N (2021) Structural analysis of glycosylglycerolipids using NMR spectroscopy. In: Plant Lipids: Methods and Protocols (Springer), pp 249–272 10.1007/978-1-0716-1362-7_14 10.1007/978-1-0716-1362-7_14 34047981

[CR68] Kragelund BB, Andersen KV, Madsen JC, Knudsen J, Poulsen FM (1993) Three-dimensional structure of the complex between acyl-coenzyme A binding protein and palmitoyl-coenzyme A. J Mol Biol 230:1260–1277. 10.1006/jmbi.1993.1240 8503960 10.1006/jmbi.1993.1240

[CR69] Krawczyk HE, Rotsch AH, Herrfurth C, Scholz P, Shomroni O, Salinas-Riester G, Feussner I, Ischebeck T (2022) Heat stress leads to rapid lipid remodeling and transcriptional adaptations in *Nicotiana tabacum* pollen tubes. Plant Physiol 189:490–515. 10.1093/plphys/kiac127 35302599 10.1093/plphys/kiac127 PMC9157110

[CR70] Ladbury JE, Klebe G, Freire E (2010) Adding calorimetric data to decision making in lead discovery: a hot tip. Nat Rev Drug Discov 9:23–27. 10.1038/nrd3054 19960014 10.1038/nrd3054

[CR71] Laha D, Kamleitner M, Johnen P, Schaaf G (2021) Analyses of inositol phosphates and phosphoinositides by strong anion exchange (SAX)-HPLC. In: Plant Lipids: Methods and Protocols (Springer), pp 365–378 10.1007/978-1-0716-1362-7_20 10.1007/978-1-0716-1362-7_20 34047987

[CR72] Leung KC, Li HY, Xiao S, Tse MH, Chye ML (2006) *Arabidopsis* ACBP3 is an extracellularly targeted acyl-CoA-binding protein. Planta 223:871–881. 10.1007/s11103-004-0642-z 16231156 10.1007/s00425-005-0139-2

[CR73] Li HY, Xiao S, Chye ML (2008) Ethylene- and pathogen-inducible *Arabidopsis* acyl-CoA-binding protein 4 interacts with an ethylene-responsive element binding protein. J Exp Bot 59:3997–4006. 10.1093/jxb/ern241 18836139 10.1093/jxb/ern241 PMC2576630

[CR74] Li N, Gügel IL, Giavalisco P, Zeisler V, Schreiber L, Soll J, Philippar K (2015) FAX1, a novel membrane protein mediating plastid fatty acid export. PLoS Biol 13:e1002053. 10.1371/journal.pbio.1002053 25646734 10.1371/journal.pbio.1002053 PMC4344464

[CR75] Li N, Xu C, Li-Beisson Y, Philippar K (2016) Fatty acid and lipid transport in plant cells. Trends Plant Sci 21:145–158. 10.1016/j.tplants.2015.10.011 26616197 10.1016/j.tplants.2015.10.011

[CR76] Li N, Meng H, Li S, Zhang Z, Zhao X, Wang S, Liu A, Li Q, Song Q, Li X (2020) Two plastid fatty acid exporters contribute to seed oil accumulation in Arabidopsis. Plant Physiol 182:1910–1919. 10.1104/pp.19.01344 32019874 10.1104/pp.19.01344 PMC7140923

[CR77] Liao P, Woodfield HK, Harwood JL, Chye ML, Scofield S (2019) Comparative transcriptomics analysis of *Brassica napus* L. during seed maturation reveals dynamic changes in gene expression between embryos and seed coats and distinct expression profiles of acyl-CoA-binding proteins for lipid accumulation. Plant Cell Physiol 60:2812–2825. 10.1093/pcp/pcz169 31504915 10.1093/pcp/pcz169 PMC6896696

[CR78] Li-Beisson Y, Shorrosh B, Beisson F, Andersson MX, Arondel V, Bates PD, Baud S, Bird D, DeBono A, Durrett TP (2013) Acyl-lipid metabolism. In the Arabidopsis Book/american Society of Plant Biologists. 10.1199/tab.0161 10.1199/tab.0161 PMC3563272 23505340

[CR79] Lu S, Sturtevant D, Aziz M, Jin C, Li Q, Chapman KD, Guo L (2018) Spatial analysis of lipid metabolites and expressed genes reveals tissue-specific heterogeneity of lipid metabolism in high-and low-oil *Brassica napus* L. seeds. Plant J 94:915–932. 10.1111/tpj.13959 29752761 10.1111/tpj.13959

[CR80] Lung SC, Liao P, Yeung EC, Hsiao AS, Xue Y, Chye ML (2017) Acyl-CoA-binding protein ACBP1 modulates sterol synthesis during embryogenesis. Plant Physiol 174:1420–1435. 10.1111/nph.14965 28500265 10.1104/pp.17.00412 PMC5490911

[CR81] Lung SC, Liao P, Yeung EC, Hsiao AS, Xue Y, Chye ML (2018) Arabidopsis ACYL-COA-BINDING PROTEIN1 interacts with STEROL C4-METHYL OXIDASE1-2 to modulate gene expression of homeodomain-leucine zipper IV transcription factors. New Phytol 218:183–200. 10.1111/nph.14965 29288621 10.1111/nph.14965

[CR82] Lung SC, Lai SH, Wang H, Zhang X, Liu A, Guo ZH, Lam HM, Chye ML (2022) Oxylipin signaling in salt-stressed soybean is modulated by ligand-dependent interaction of Class II acyl-CoA-binding proteins with lipoxygenase. Plant Cell 34:1117–1143. 10.1093/plcell/koab306 34919703 10.1093/plcell/koab306 PMC8894927

[CR83] Ma S, Leng Y, Li X, Meng Y, Yin Z, Hang W (2023) High spatial resolution mass spectrometry imaging for spatial metabolomics: Advances, challenges, and future perspectives. TrAC, Trends Anal. Chem. 159:116902 10.1016/j.trac.2022.116902

[CR84] Marmon S, Sturtevant D, Herrfurth C, Chapman K, Stymne S, Feussner I (2017) Two acyltransferases contribute differently to linolenic acid levels in seed oil. Plant Physiol 173:2081–2095. 10.1104/pp.16.01865 28235891 10.1104/pp.16.01865 PMC5373062

[CR85] McNair HM, Miller JM, Snow NH (2019) Basic gas chromatography. (John Wiley & Sons).

[CR86] Men X, Shi J, Liang W, Zhang Q, Lian G, Quan S, Zhu L, Luo Z, Chen M, Zhang D (2017) Glycerol-3-Phosphate Acyltransferase 3 (OsGPAT3) is required for anther development and male fertility in rice. J Exp Bot 68:513–526. 10.1093/jxb/erw445 28082511 10.1093/jxb/erw445 PMC6055571

[CR87] Meng W, Su YCF, Saunders RMK, Chye ML (2011) The rice acyl-CoA-binding protein gene family: phylogeny, expression and functional analysis. New Phytol 189:1170–1184. 10.1111/j.1469-8137.2010.03546.x 21128943 10.1111/j.1469-8137.2010.03546.x

[CR88] Miao R, Lung SC, Li X, Li XD, Chye ML (2019) Thermodynamic insights into an interaction between ACYL-CoA–BINDING PROTEIN2 and LYSOPHOSPHOLIPASE2 in Arabidopsis. J Biol Chem 294:6214–6226. 10.1074/jbc.RA118.006876 30782848 10.1074/jbc.RA118.006876 PMC6484133

[CR89] Montini L, Crocoll C, Gleadow RM, Motawia MS, Janfelt C, Bjarnholt N (2020) Matrix-assisted laser desorption/ionization-mass spectrometry imaging of metabolites during sorghum germination. Plant Physiol 183:925–942. 10.1104/pp.19.01357 32350122 10.1104/pp.19.01357 PMC7333723

[CR90] Monzani PS, Pereira HM, Melo FA, Meirelles FV, Oliva G, Cascardo JCM (2010) A new topology of ACBP from Moniliophthora perniciosa. Biochim. Biophys Acta, Proteins Proteomics 1804:115–123. 10.1016/j.bbapap.2009.09.020 10.1016/j.bbapap.2009.09.020 19782157

[CR91] Munir R, Semmar N, Farman M, Ahmad NS (2017) An updated review on pharmacological activities and phytochemical constituents of evening primrose (genus *Oenothera*). Asian Pac J Trop Biomed 7:1046–1054. 10.1016/j.apjtb.2017.10.004

[CR92] Newton AC, Bootman MD, Scott JD (2016) Second Messengers CSH Perspect Biol 8:a005926. 10.1101/cshperspect.a005926 10.1101/cshperspect.a005926 PMC4968160 27481708

[CR93] Ohlrogge J, Browse J (1995) Lipid biosynthesis. Plant Cell 7:957–970 7640528 10.1105/tpc.7.7.957 PMC160893

[CR94] Ortiz R, Geleta M, Gustafsson C, Lager I, Hofvander P, Löfstedt C, Cahoon EB, Minina E, Bozhkov P, Stymne S (2020) Oil crops for the future. Curr Opin Plant Biol 56:181–189. 10.1016/j.pbi.2019.12.003 31982290 10.1016/j.pbi.2019.12.003

[CR95] Oubohssaine M, Hnini M, Rabeh K (2024) Exploring lipid signaling in plant physiology: from cellular membranes to environmental adaptation. J Plant Physiol 300:154295. 10.1016/j.jplph.2024.154295 38885581 10.1016/j.jplph.2024.154295

[CR96] Owen HA, Makaroff C (1995) Ultrastructure of microsporogenesis and microgametogenesis in *Arabidopsis thaliana* (L.) Heynh. ecotype *Wassilewskija* (Brassicaceae). Protoplasma 185:7–21. 10.1007/BF01272749

[CR97] Ozseyhan ME, Li P, Na G, Li Z, Wang C, Lu C (2018) Improved fatty acid profiles in seeds of *Camelina sativa* by artificial microRNA mediated FATB gene suppression. Biochem Biophys Res Comm 503:621–624. 10.1016/j.bbrc.2018.06.051 29906463 10.1016/j.bbrc.2018.06.051

[CR98] Pajoro A, Biewers S, Dougali E, Leal Valentim F, Mendes MA, Porri A, Coupland G, Van de Peer Y, Van Dijk AD, Colombo L (2014) The (r)evolution of gene regulatory networks controlling Arabidopsis plant reproduction: a two-decade history. J Exp Bot 65:4731–4745. 10.1093/jxb/eru233 24913630 10.1093/jxb/eru233

[CR99] Parakkunnel R, Naik BN, Vanishree G, George A, Kv S, Yr A, K UB, Anandan A, Kumar S (2024) Exploring selection signatures in the divergence and evolution of lipid droplet (LD) associated genes in major oilseed crops. BMC Genomics 25, 653 10.1186/s12864-024-10527-4 10.1186/s12864-024-10527-4 PMC11218257 38956471

[CR100] Pastor S, Sethumadhavan K, Ullah AH, Gidda S, Cao H, Mason C, Chapital D, Scheffler B, Mullen R, Dyer J (2013) Molecular properties of the class III subfamily of acyl-coenyzme A binding proteins from tung tree (Vernicia fordii). Plant Sci 203:79–88. 10.1016/j.plantsci.2012.12.009 23415331 10.1016/j.plantsci.2012.12.009

[CR101] Pati S, Nie B, Arnold RD, Cummings BS (2016) Extraction, chromatographic and mass spectrometric methods for lipid analysis. Biomed Chromatogr 30:695–709. 10.1002/bmc.3683 26762903 10.1002/bmc.3683 PMC8425715

[CR102] Piffanelli P, Ross JH, Murphy DJ (1997) Intra-and extracellular lipid composition and associated gene expression patterns during pollen development in *Brassica napus*. Plant J 11:549–562. 10.1007/s004970050122 9107041 10.1046/j.1365-313x.1997.11030549.x

[CR103] Piffanelli P, Ross JH, Murphy D (1998) Biogenesis and function of the lipidic structures of pollen grains. Sex Plant Reprod 11:65–80. 10.1007/s004970050122

[CR104] Qin H, Li H, Abhinandan K, Xun B, Yao K, Shi J, Zhao R, Li M, Wu Y, Lan X (2022) Fatty acid biosynthesis pathways are downregulated during stigma development and are critical during self-incompatible responses in ornamental kale. Int J Mol Sci 23:13102. 10.3390/ijms232113102 36361887 10.3390/ijms232113102 PMC9656282

[CR105] Quilichini TD, Friedmann MC, Samuels AL, Douglas CJ (2010) ATP-binding cassette transporter G26 is required for male fertility and pollen exine formation in Arabidopsis. Plant Physiol 154:678–690. 10.1104/pp.110.161968 20732973 10.1104/pp.110.161968 PMC2949020

[CR106] Raboanatahiry N, Wang B, Yu L, Li M (2018) Functional and structural diversity of acyl-CoA binding proteins in oil crops. Front Genet 9:373083. 10.3389/fgene.2018.00182 10.3389/fgene.2018.00182 PMC5972291 29872448

[CR107] Rasmussen J, Börchers T, Knudsen J (1990) Comparison of the binding affinities of acyl-CoA-binding protein and fatty-acid-binding protein for long-chain acyl-CoA esters. Biochem J 265:849–855. 10.1042/bj2650849 2306218 10.1042/bj2650849 PMC1133709

[CR108] Raygan F, Taghizadeh M, Mirhosseini N, Akbari E, Bahmani F, Memarzadeh MR, Sharifi N, Jafarnejad S, Banikazemi Z, Asemi Z (2019) A comparison between the effects of flaxseed oil and fish oil supplementation on cardiovascular health in type 2 diabetic patients with coronary heart disease: a randomized, double-blinded, placebo-controlled trial. Phytother Res 33:1943–1951. 10.1002/ptr.6393 31190359 10.1002/ptr.6393

[CR109] Rotsch AH, Kopka J, Feussner I, Ischebeck T (2017) Central metabolite and sterol profiling divides tobacco male gametophyte development and pollen tube growth into eight metabolic phases. Plant J 92:129–146. 10.1111/tpj.13633 28685881 10.1111/tpj.13633

[CR110] Saini RK, Prasad P, Shang X, Keum YS (2021) Advances in lipid extraction methods—a review. Int J Mol Sci 22:13643. 10.3390/ijms222413643 34948437 10.3390/ijms222413643 PMC8704327

[CR111] Shi J, Cui M, Yang L, Kim YJ, Zhang D (2015) Genetic and biochemical mechanisms of pollen wall development. Trends Plant Sci 20:741–753. 10.1016/j.tplants.2015.07.010 26442683 10.1016/j.tplants.2015.07.010

[CR112] Song J, Mavraganis I, Shen W, Yang H, Patterson N, Wang L, Xiang D, Cui Y, Zou J (2024) Pistil-derived lipids influence pollen tube growth and male fertility in *Arabidopsis thaliana*. Plant Physiol 196:763–772. 10.1093/plphys/kiae276 38917229 10.1093/plphys/kiae276

[CR113] Stevenson JM, Perera IY, Boss WF (1998) A phosphatidylinositol 4-kinase pleckstrin homology domain that binds phosphatidylinositol 4-monophosphate. J Biol Chem 273:22761–22767. 10.1074/jbc.273.35.22761 9712908 10.1074/jbc.273.35.22761

[CR114] Sturtevant D, Dueñas ME, Lee YJ, Chapman KD (2017a) Three-dimensional visualization of membrane phospholipid distributions in *Arabidopsis thaliana* seeds: A spatial perspective of molecular heterogeneity. Biochim. Biophys. Acta. Mol Cell Biol Lipid 1862:268–281. 10.1016/j.bbalip.2016.11.012 10.1016/j.bbalip.2016.11.012 27919665

[CR115] Sturtevant D, Horn P, Kennedy C, Hinze L, Percy R, Chapman K (2017b) Lipid metabolites in seeds of diverse *Gossypium* accessions: molecular identification of a high oleic mutant allele. Planta 245:595–610. 10.1007/s00425-016-2630-3 27988885 10.1007/s00425-016-2630-3

[CR116] Sturtevant D, Aziz M, Romsdahl TB, Corley CD, Chapman KD (2021) In situ localization of plant lipid metabolites by matrix-assisted laser desorption/ionization mass spectrometry imaging (MALDI-MSI). In: Plant Lipids: Methods and Protocols (Springer), pp 417–438 10.1007/978-1-0716-1362-7_24 10.1007/978-1-0716-1362-7_24 34047991

[CR117] Sumara A, Stachniuk A, Montowska M, Kotecka-Majchrzak K, Grywalska E, Mitura P, SaftićMartinović L, KraljevićPavelić S, Fornal E (2023) Comprehensive review of seven plant seed oils: chemical composition, nutritional properties, and biomedical functions. Food Rev Int 39:5402–5422. 10.1080/87559129.2022.2067560

[CR118] Talamonti E, Sasso V, To H, Haslam R, Napier JA, Ulfhake B, Pernold K, Asadi A, Hessa T, Jacobsson A (2020) Impairment of DHA synthesis alters the expression of neuronal plasticity markers and the brain inflammatory status in mice. FASEB J 34:2024–2040. 10.1096/fj.201901890RR 31909582 10.1096/fj.201901890RR PMC7384056

[CR119] Taskinen JP, van Aalten DM, Knudsen J, Wierenga RK (2007) High resolution crystal structures of unliganded and liganded human liver ACBP reveal a new mode of binding for the acyl-CoA ligand. Protein Struct Funct Bioinfo 66:229–238. 10.1002/prot.21124 10.1002/prot.21124 17044054

[CR120] Teilum K, Thormann T, Caterer NR, Poulsen HI, Jensen PH, Knudsen J, Kragelund BB, Poulsen FM (2005) Different secondary structure elements as scaffolds for protein folding transition states of two homologous four-helix bundles. Protein Struct Funct Bioinfo 59:80–90. 10.1002/prot.20340 10.1002/prot.20340 15690348

[CR121] Troncoso-Ponce MA, Nikovics K, Marchive C, Lepiniec L, Baud S (2016) New insights on the organization and regulation of the fatty acid biosynthetic network in the model higher plant *Arabidopsis thaliana*. Biochimie 120:3–8. 10.1016/j.biochi.2015.05.013 26025475 10.1016/j.biochi.2015.05.013

[CR122] Usher S, Han L, Haslam RP, Michaelson LV, Sturtevant D, Aziz M, Chapman KD, Sayanova O, Napier JA (2017) Tailoring seed oil composition in the real world: optimising omega-3 long chain polyunsaturated fatty acid accumulation in transgenic *Camelina sativa*. Sci Rep 7:6570. 10.1038/s41598-017-06838-0 28747792 10.1038/s41598-017-06838-0 PMC5529437

[CR123] Vallarino JG, Jun H, Wang S, Wang X, Sade N, Orf I, Zhang D, Shi J, Shen S, Cuadros-Inostroza Á (2023) Limitations and advantages of using metabolite-based genome-wide association studies: Focus on fruit quality traits. Plant Sci. 10.1016/j.plantsci.2023.111748 37230189 10.1016/j.plantsci.2023.111748

[CR124] van Aalten DMF, Milne KG, Zou JY, Kleywegt GJ, Bergfors T, Ferguson MAJ, Knudsen J, Jones TA (2001) Binding site differences revealed by crystal structures of *Plasmodium falciparum* and bovine acyl-CoA binding protein. J Mol Biol 309:181–192. 10.1006/jmbi.2001.4749 11491287 10.1006/jmbi.2001.4749

[CR125] Wan X, Wu S, Li Z, An X, Tian Y (2020) Lipid metabolism: critical roles in male fertility and other aspects of reproductive development in plants. Mol Plant 13:955–983. 10.1016/j.molp.2020.05.009 32434071 10.1016/j.molp.2020.05.009

[CR126] Wang HYJ, Hsu FF (2020) Revelation of acyl double bond positions on fatty acyl coenzyme A esters by MALDI/TOF mass spectrometry. JASMS 31:1047–1057. 10.1021/jasms.9b00139 10.1021/jasms.9b00139 32167298

[CR127] Wang X, Chen Y, Liu Y, Ouyang L, Yao R, Wang Z, Kang Y, Yan L, Huai D, Jiang H (2022) Visualizing the distribution of lipids in peanut seeds by MALDI mass spectrometric imaging. Foods 11:3888. 10.3390/foods11233888 36496696 10.3390/foods11233888 PMC9739101

[CR128] Wattelet-Boyer V, Brocard L, Jonsson K, Esnay N, Joubès J, Domergue F, Mongrand S, Raikhel N, Bhalerao RP, Moreau P (2016) Enrichment of hydroxylated C24-and C26-acyl-chain sphingolipids mediates PIN2 apical sorting at trans-Golgi network subdomains. Nat Commun 7:12788. 10.1038/ncomms12788 27681606 10.1038/ncomms12788 PMC5056404

[CR129] Wiseman T, Williston S, Brandts JF, Lin LN (1989) Rapid measurement of binding constants and heats of binding using a new titration calorimeter. Anal Biochem 179:131–137. 10.1016/0003-2697(89)90213-3 2757186 10.1016/0003-2697(89)90213-3

[CR130] Wolters-Arts M, Lush WM, Mariani C (1998) Lipids are required for directional pollen-tube growth. Nature 392:818–821. 10.1038/33929 9572141 10.1038/33929

[CR131] Woodfield HK, Sturtevant D, Borisjuk L, Munz E, Guschina IA, Chapman K, Harwood JL (2017) Spatial and temporal mapping of key lipid species in *Brassica napus* seeds. Plant Physiol 173:1998–2009. 10.1104/pp.16.01705 28188274 10.1104/pp.16.01705 PMC5373056

[CR132] Xiao S, Chye ML (2011) Overexpression of Arabidopsis ACBP3 enhances NPR1-dependent plant resistance to *Pseudomonas syringe* pv *tomato* DC3000. Plant Physiol 156:2069–2081. 10.1104/pp.111.176933 21670223 10.1104/pp.111.176933 PMC3149925

[CR133] Xiao C, Jingshi X, Zhongnan Y (2025) Studies on the composition and structure of plant sporopollenin. Chinese Journal of Nature, 1–8.

[CR134] Xu D, Shi J, Rautengarten C, Yang L, Qian X, Uzair M, Zhu L, Luo Q, An G, Waßmann F (2017) *Defective Pollen Wall 2 (DPW2)* encodes an acyl transferase required for rice pollen development. Plant Physiol 173:240–255. 10.1104/pp.16.00095 27246096 10.1104/pp.16.00095 PMC5210703

[CR135] Xue Y, Xiao S, Kim J, Lung SC, Chen L, Tanner JA, Suh MC, Chye ML (2014) Arabidopsis membrane-associated acyl-CoA-binding protein ACBP1 is involved in stem cuticle formation. J Exp Bot 65:5473–5483. 10.1093/jxb/eru304 25053648 10.1093/jxb/eru304 PMC4157719

[CR136] Yadava P, Singh A, Kumar K, Singh I (2019) Plant senescence and agriculture. In: Senescence signalling and control in plants, pp. 283–302.

[CR137] Yang X, Liang W, Chen M, Zhang D, Zhao X, Shi J (2017) Rice fatty acyl-CoA synthetase OsACOS12 is required for tapetum programmed cell death and male fertility. Planta 246:105–122. 10.1007/s00425-017-2691-y 28382520 10.1007/s00425-017-2691-y

[CR138] Yang R, Zhang L, Li P, Yu L, Mao J, Wang X, Zhang Q (2018) A review of chemical composition and nutritional properties of minor vegetable oils in China. Trends Food Sci Tech 74:26–32. 10.1016/j.tifs.2018.01.013

[CR139] Yao X, Hu W, Yang ZN (2022) The contributions of sporophytic tapetum to pollen formation. Seed Biol. 1:1–13. 10.48130/SeedBio-2022-0005

[CR140] Ye ZW, Chen QF, Chye ML (2017a) *Arabidopsis thaliana* acyl-CoA-binding protein ACBP6 interacts with plasmodesmata-located protein PDLP8. Plant Signal Behav 12:e1359365. 10.1080/15592324.2017.1359365 28786767 10.1080/15592324.2017.1359365 PMC5616145

[CR141] Ye ZW, Xu J, Shi J, Zhang D, Chye ML (2017b) Kelch-motif containing acyl-CoA binding proteins AtACBP4 and AtACBP5 are differentially expressed and function in floral lipid metabolism. Plant Mol Biol 93:209–225. 10.1007/s11103-016-0557-5 27826761 10.1007/s11103-016-0557-5

[CR142] Yin Z, Huang W, Fernie AR, Yan S (2023) Mass spectrometry imaging techniques: a versatile toolbox for plant metabolomics. Trends Plant Sci 28:250–251. 10.1016/j.tplants.2022.10.009 36411181 10.1016/j.tplants.2022.10.009

[CR143] Yuan H, Jiangfang Y, Liu Z, Su R, Li Q, Fang C, Huang S, Liu X, Fernie AR, Luo J (2024) WTV2. 0: A high-coverage plant volatilomics method with a comprehensive selective ion monitoring acquisition mode. Mol Plant 17:972–985. 10.1016/j.molp.2024.04.012 38685707 10.1016/j.molp.2024.04.012

[CR144] Yurchenko O, Singer SD, Nykiforuk CL, Gidda S, Mullen RT, Moloney MM, Weselake RJ (2014) Production of a *Brassica napus* low-molecular mass acyl-coenzyme A-binding protein in Arabidopsis alters the acyl-coenzyme A pool and acyl composition of oil in seeds. Plant Physiol 165:550–560. 10.1104/pp.114.238071 24740000 10.1104/pp.114.238071 PMC4044837

[CR145] Zamora Obando HR, Duarte GHB, Simionato AVC (2021) Metabolomics data treatment: basic directions of the full process. In: Separation Techniques Applied to Omics Sciences: From Principles to Relevant Applications, pp 243–264 10.1007/978-3-030-77252-9_12 34628635

[CR146] Zhan H, Xiong H, Wang S, Yang ZN (2018) Anther endothecium-derived very-long-chain fatty acids facilitate pollen hydration in Arabidopsis. Mol Plant 11:1101–1104. 10.1016/j.molp.2018.05.002 29763723 10.1016/j.molp.2018.05.002

[CR147] Zhang M, Fan J, Taylor DC, Ohlrogge JB (2009) DGAT1 and PDAT1 acyltransferases have overlapping functions in Arabidopsis triacylglycerol biosynthesis and are essential for normal pollen and seed development. Plant Cell 21:3885–3901. 10.1105/tpc.109.071795 20040537 10.1105/tpc.109.071795 PMC2814504

[CR148] Zhang D, Liang W, Yin C, Zong J, Gu F, Zhang D (2010) *OsC6*, encoding a lipid transfer protein, is required for postmeiotic anther development in rice. Plant Physiol 154:149–162. 10.1104/pp.110.158865 20610705 10.1104/pp.110.158865 PMC2938136

[CR149] Zhang M, Kim Y, Zong J, Lin H, Dievart A, Li H, Zhang D, Liang W (2019) Genome-wide analysis of the barley non-specific lipid transfer protein gene family. Crop J 7:65–76. 10.1186/s12864-021-07958-8

[CR150] Zhang S, Wu S, Niu C, Liu D, Yan T, Tian Y, Liu S, Xie K, Li Z, Wang Y (2021a) *ZmMs25* encoding a plastid-localized fatty acyl reductase is critical for anther and pollen development in maize. J Exp Bot 72:4298–4318. 10.1093/jxb/erab142 33822021 10.1093/jxb/erab142

[CR151] Zhang Z, Zhan H, Lu J, Xiong S, Yang N, Yuan H, Yang Z-N (2021b) Tapetal 3-Ketoacyl-Coenzyme A synthases are involved in pollen coat lipid accumulation for pollen-stigma interaction in Arabidopsis. Front Plant Sci 12:770311. 10.3389/fpls.2021.770311 34887893 10.3389/fpls.2021.770311 PMC8650583

[CR152] Zhang D, Shi J, Yang X (2016) Role of lipid metabolism in plant pollen exine development. In: Lipids in plant and algae development pp 315 337 10.1007/978-3-319-25979-6_13 27023241

[CR153] Zhao L, Katavic V, Li F, Haughn GW, Kunst L (2010) Insertional mutant analysis reveals that long-chain acyl-CoA synthetase 1 (LACS1), but not LACS8, functionally overlaps with LACS9 in Arabidopsis seed oil biosynthesis. Plant J 64:1048–1058. 10.1111/j.1365-313X.2010.04396.x 21143684 10.1111/j.1365-313X.2010.04396.x

[CR154] Zhao J, Bi R, Li S, Zhou D, Bai Y, Jing G, Zhang K, Zhang W (2019) Genome-wide analysis and functional characterization of Acyl-CoA:diacylglycerol acyltransferase from soybean identify GmDGAT1A and 1B roles in oil synthesis in Arabidopsis seeds. J Plant Physiol 242:153019. 10.1016/j.jplph.2019.153019 31437808 10.1016/j.jplph.2019.153019

[CR155] Zhao W, Hou Q, Qi Y, Wu S, Wan X (2023) Structural and molecular basis of pollen germination. Plant Physiol Biochem 203:108042. 10.1016/j.plaphy.2023.108042 37738868 10.1016/j.plaphy.2023.108042

[CR156] Zheng H, Rowland O, Kunst L (2005) Disruptions of the Arabidopsis enoyl-CoA reductase gene reveal an essential role for very-long-chain fatty acid synthesis in cell expansion during plant morphogenesis. Plant Cell 17:1467–1481. 10.1105/tpc.104.030155 15829606 10.1105/tpc.104.030155 PMC1091768

[CR157] Zhu K, Li N, Zheng X, Sarwar R, Li Y, Cao J, Wang Z, Tan X (2023) Overexpression the BnLACS9 could increase the chlorophyll and oil content in *Brassica napus*. Biotechnol Biofuels Bioprod 16:3. 10.1186/s13068-022-02254-3 36609294 10.1186/s13068-022-02254-3 PMC9825004

[CR158] Zienkiewicz A, Zienkiewicz K, Rejón JD, Rodríguez-García MI, Castro AJ (2013) New insights into the early steps of oil body mobilization during pollen germination. J Exp Bot 64:293–302. 10.1093/jxb/ers332 23132905 10.1093/jxb/ers332 PMC3528035

[CR159] Zolman BK, Silva ID, Bartel B (2001) The Arabidopsis *pxa1* mutant is defective in an ATP-binding cassette transporter-like protein required for peroxisomal fatty acid β-oxidation. Plant Physiol 127:1266–1278 11706205 PMC129294

[CR160] Zu P, Koch H, Schwery O, Pironon S, Phillips C, Ondo I, Farrell IW, Nes WD, Moore E, Wright GA (2021) Pollen sterols are associated with phylogeny and environment but not with pollinator guilds. New Phytol 230:1169–1184. 10.1111/nph.17227 33484583 10.1111/nph.17227 PMC8653887

